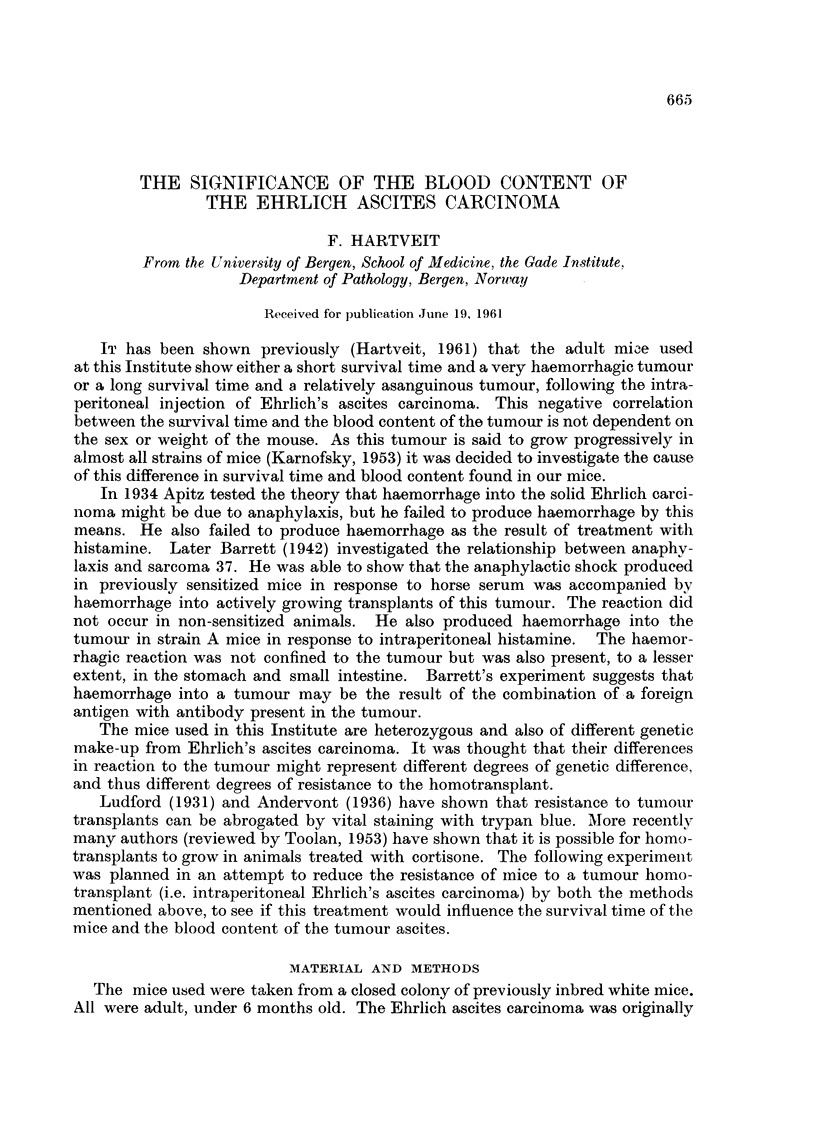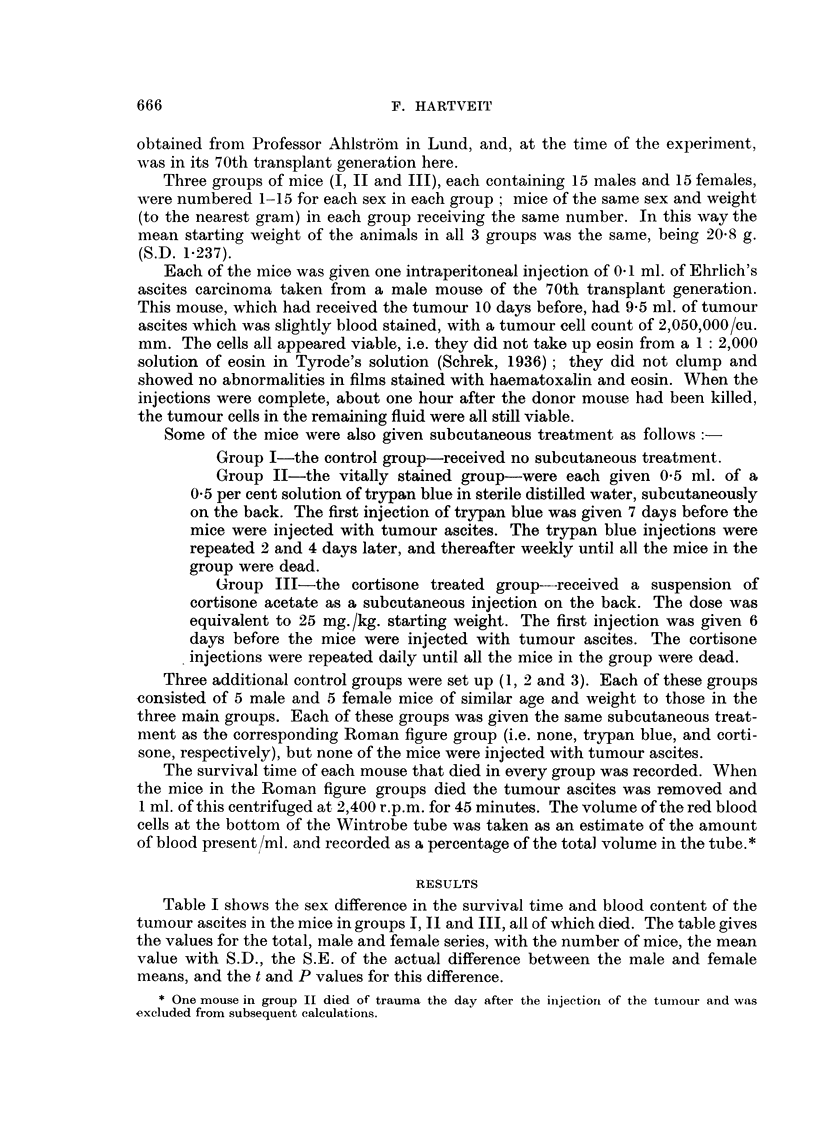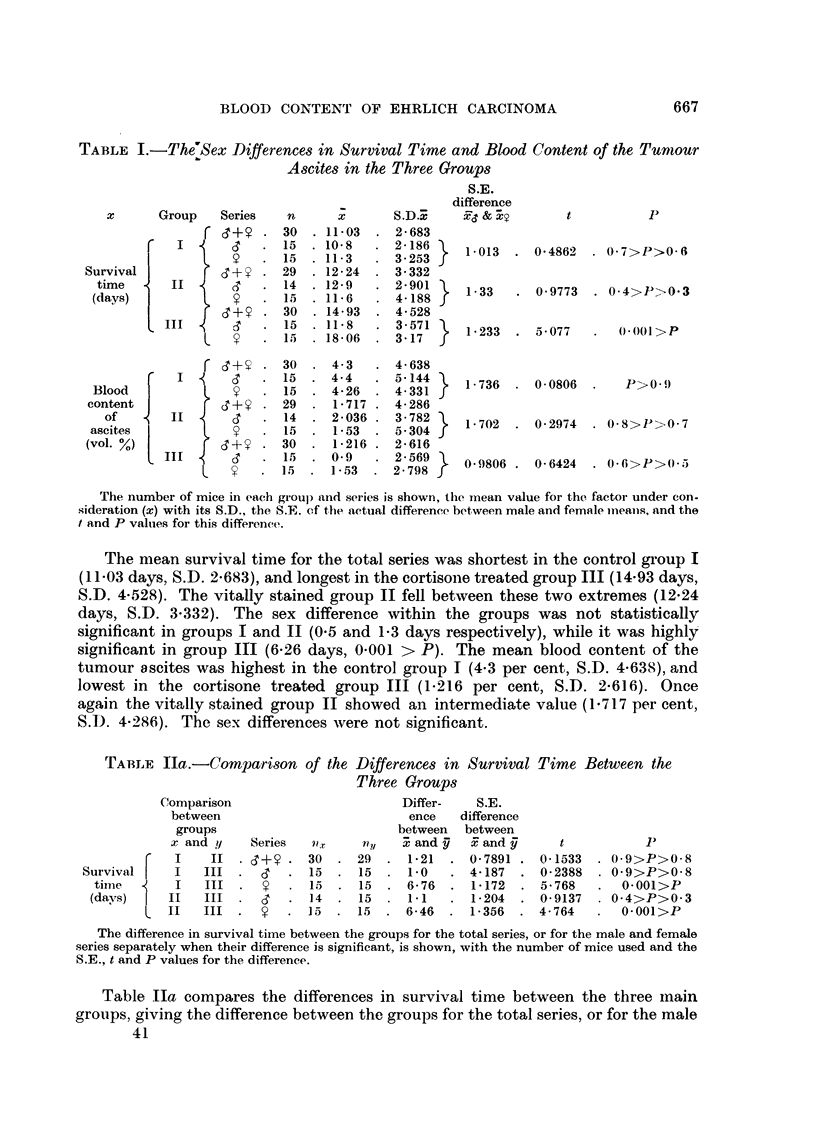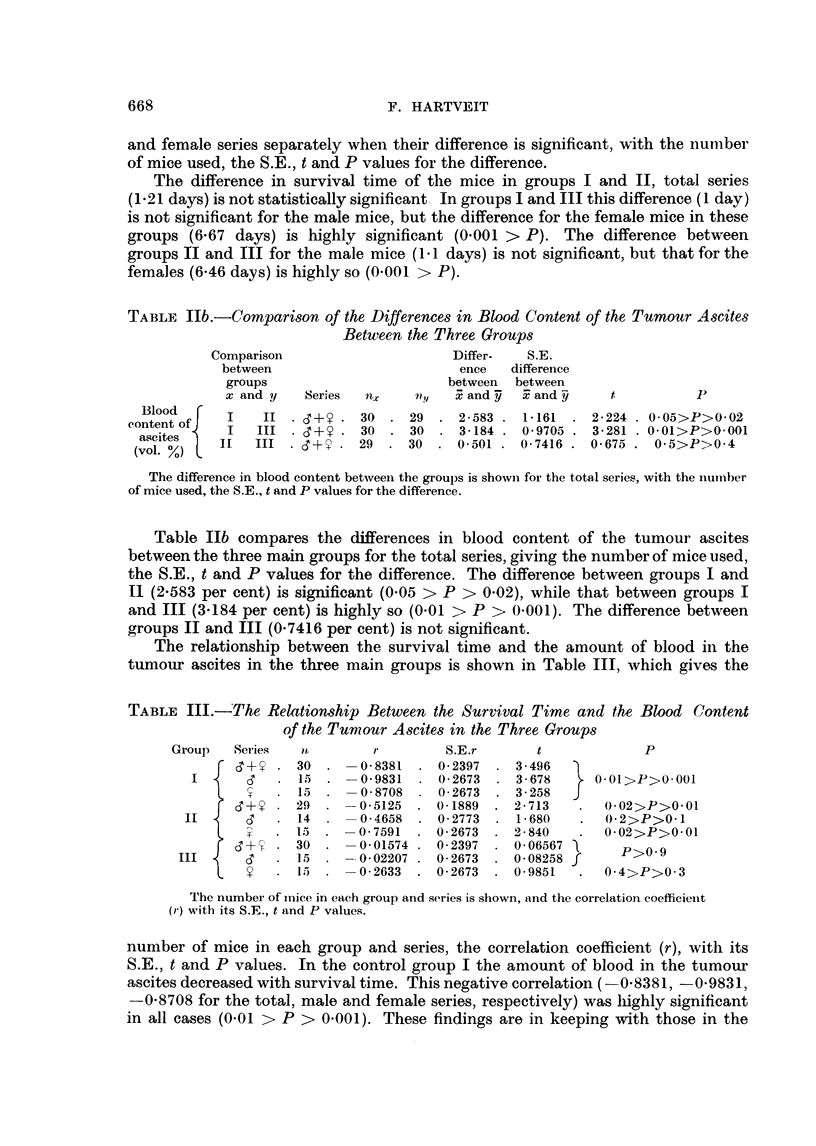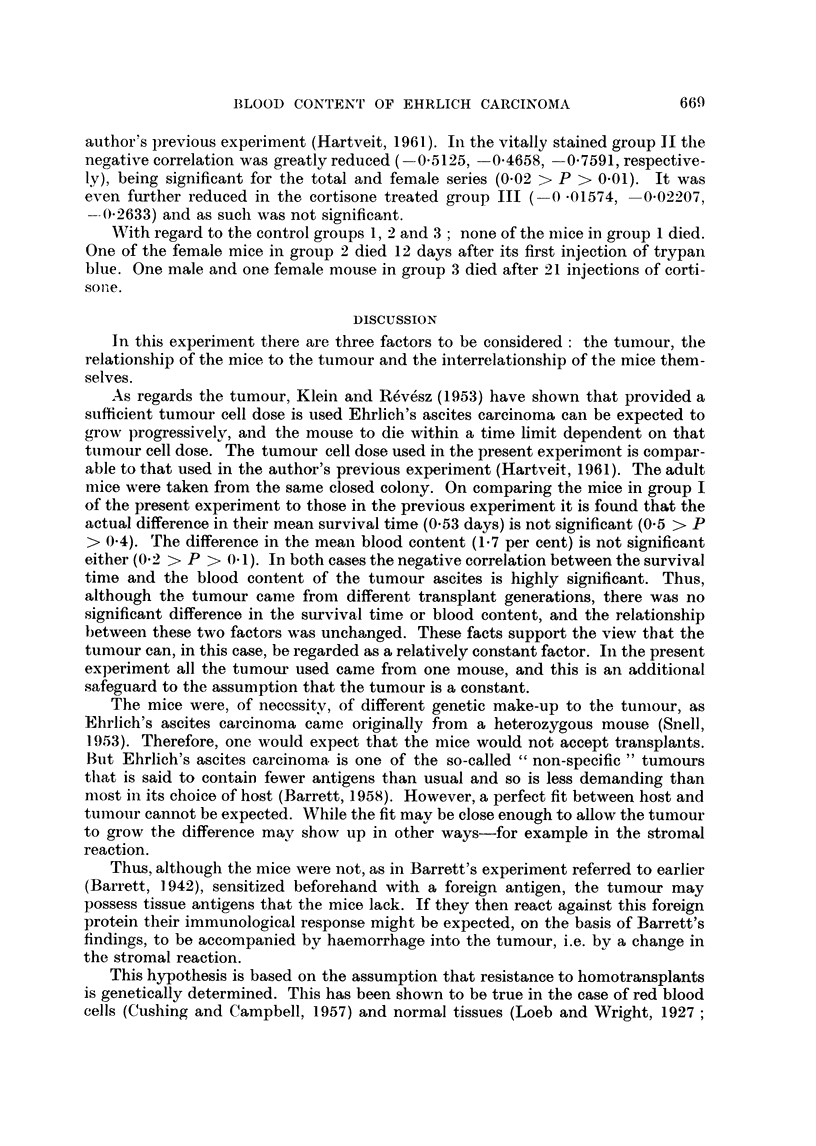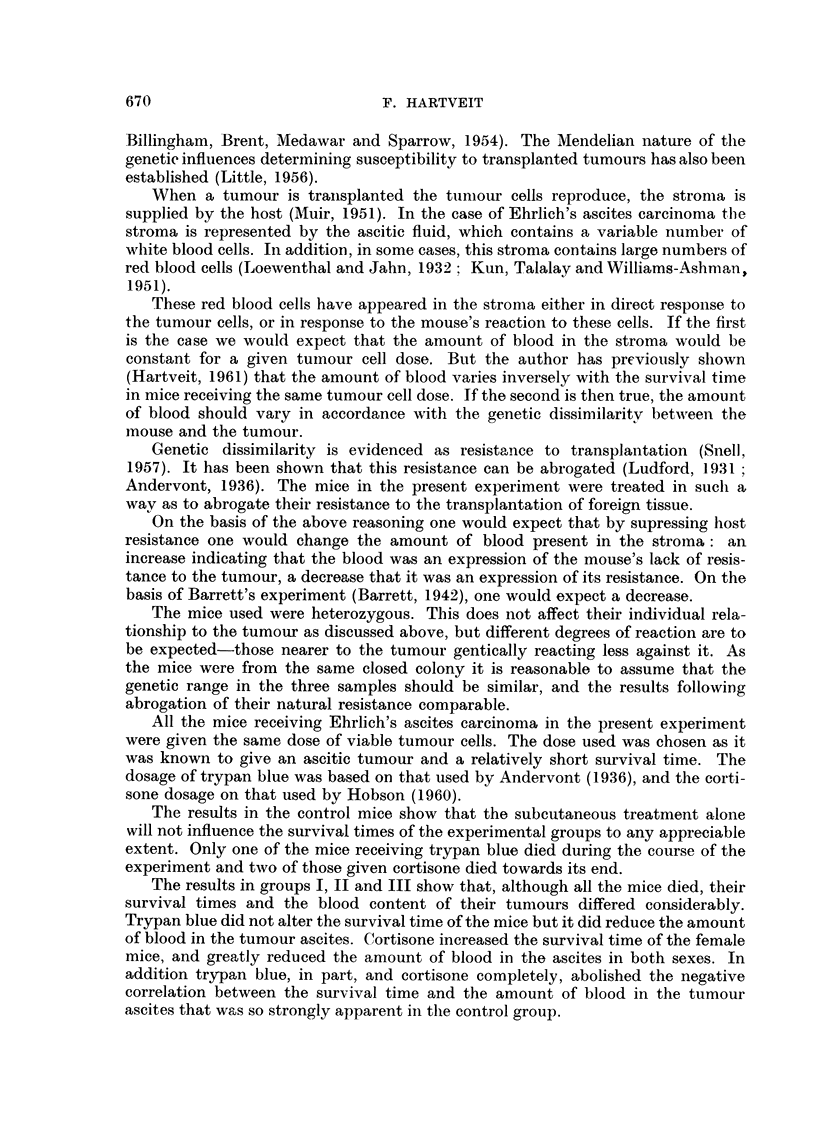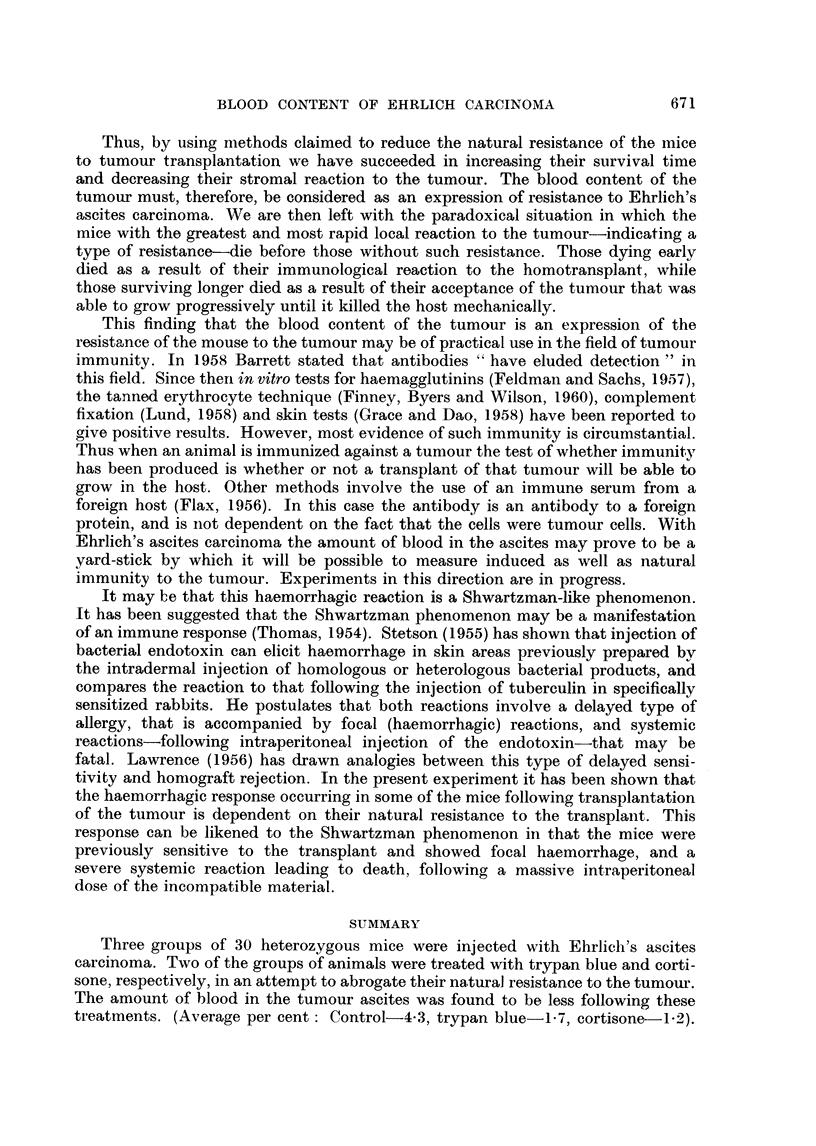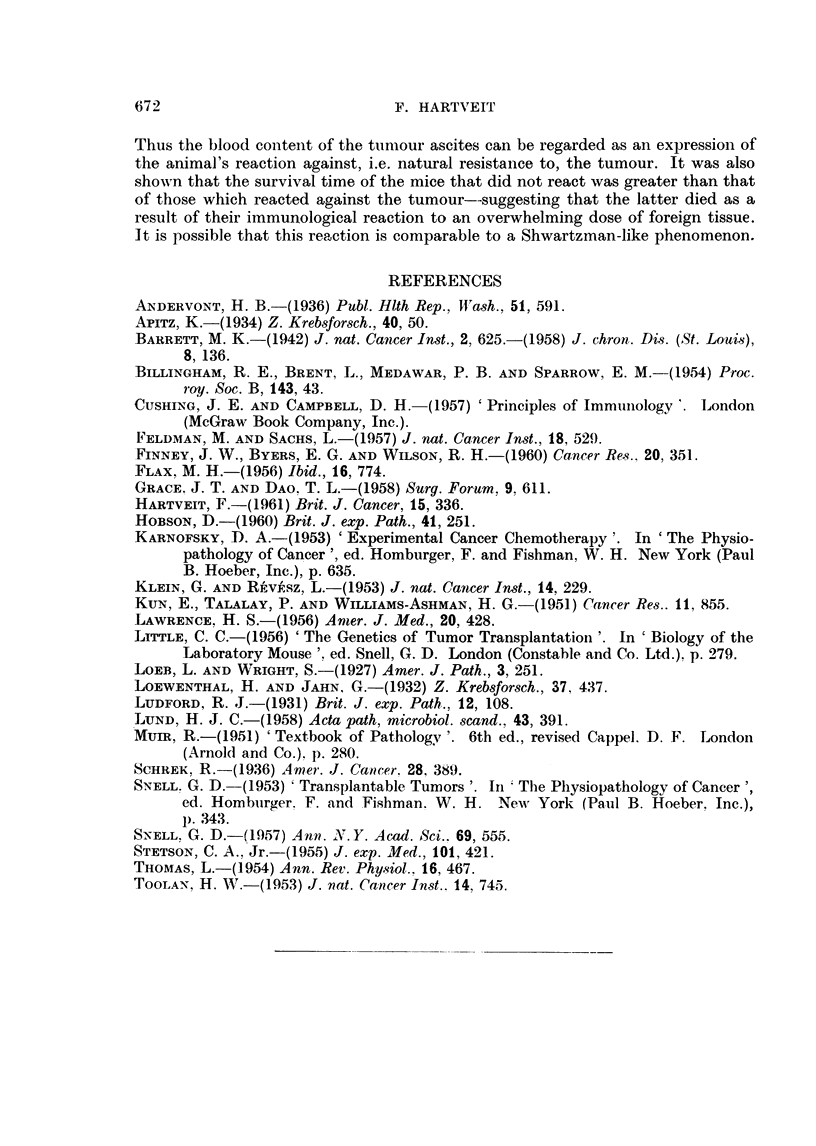# The Significance of the Blood Content of the Ehrlich Ascites Carcinoma

**DOI:** 10.1038/bjc.1961.75

**Published:** 1961-09

**Authors:** F. Hartveit


### THE SIGNIFICANCE OF THE BLOOD CONTENT OF THE EHRLICH ASCITES CARCINOMA

F. HARTVEIT

*From the University of Bergen, School of Medicine, the Gade Institute, Department of Pathology, Bergen, Norway*

Received for publication June 19, 1961

It has been shown previously (Hartveit, 1961) that the adult mice used at this Institute show either a short survival time and a very haemorrhagic tumour or a long survival time and a relatively asanguinous tumour, following the intraperitoneal injection of Ehrlich's ascites carcinoma. This negative correlation between the survival time and the blood content of the tumour is not dependent on the sex or weight of the mouse. As this tumour is said to grow progressively in almost all strains of mice (Karnofsky, 1953) it was decided to investigate the cause of this difference in survival time and blood content found in our mice.

In 1934 Apitz tested the theory that haemorrhage into the solid Ehrlich carcinoma might be due to anaphylaxis, but he failed to produce haemorrhage by this means. He also failed to produce haemorrhage as the result of treatment with histamine. Later Barrett (1942) investigated the relationship between anaphylaxis and sarcoma 37. He was able to show that the anaphylactic shock produced in previously sensitized mice in response to horse serum was accompanied by haemorrhage into actively growing transplants of this tumour. The reaction did not occur in non-sensitized animals. He also produced haemorrhage into the tumour in strain A mice in response to intraperitoneal histamine. The haemorrhagic reaction was not confined to the tumour but was also present, to a lesser extent, in the stomach and small intestine. Barrett's experiment suggests that haemorrhage into a tumour may be the result of the combination of a foreign antigen with antibody present in the tumour.

The mice used in this Institute are heterozygous and also of different genetic make-up from Ehrlich's ascites carcinoma. It was thought that their differences in reaction to the tumour might represent different degrees of genetic difference, and thus different degrees of resistance to the homotransplant.

Ludford (1931) and Andervont (1936) have shown that resistance to tumour transplants can be abrogated by vital staining with trypan blue. More recently many authors (reviewed by Toolan, 1953) have shown that it is possible for homotransplants to grow in animals treated with cortisone. The following experiment was planned in an attempt to reduce the resistance of mice to a tumour homotransplant (i.e. intraperitoneal Ehrlich's ascites carcinoma) by both the methods mentioned above, to see if this treatment would influence the survival time of the mice and the blood content of the tumour ascites.

### MATERIAL AND METHODS

The mice used were taken from a closed colony of previously inbred white mice. All were adult, under 6 months old. The Ehrlich ascites carcinoma was originally obtained from Professor Ahlström in Lund, and, at the time of the experiment, was in its 70th transplant generation here.

Three groups of mice (I, II and III), each containing 15 males and 15 females, were numbered 1–15 for each sex in each group ; mice of the same sex and weight (to the nearest gram) in each group receiving the same number. In this way the mean starting weight of the animals in all 3 groups was the same, being 20·8 g. (S.D. 1·237).

Each of the mice was given one intraperitoneal injection of 0·1 ml. of Ehrlich's ascites carcinoma taken from a male mouse of the 70th transplant generation. This mouse, which had received the tumour 10 days before, had 9·5 ml. of tumour ascites which was slightly blood stained, with a tumour cell count of 2,050,000/cu. mm. The cells all appeared viable, i.e. they did not take up eosin from a 1 : 2,000 solution of eosin in Tyrode's solution (Schrek, 1936) ; they did not clump and showed no abnormalities in films stained with haematoxalin and eosin. When the injections were complete, about one hour after the donor mouse had been killed, the tumour cells in the remaining fluid were all still viable.

Some of the mice were also given subcutaneous treatment as follows :—

Group I—the control group—received no subcutaneous treatment.

Group II—the vitally stained group—were each given 0·5 ml. of a 0·5 per cent solution of trypan blue in sterile distilled water, subcutaneously on the back. The first injection of trypan blue was given 7 days before the mice were injected with tumour ascites. The trypan blue injections were repeated 2 and 4 days later, and thereafter weekly until all the mice in the group were dead.

Group III—the cortisone treated group—received a suspension of cortisone acetate as a subcutaneous injection on the back. The dose was equivalent to 25 mg./kg. starting weight. The first injection was given 6 days before the mice were injected with tumour ascites. The cortisone injections were repeated daily until all the mice in the group were dead.

Three additional control groups were set up (1, 2 and 3). Each of these groups consisted of 5 male and 5 female mice of similar age and weight to those in the three main groups. Each of these groups was given the same subcutaneous treatment as the corresponding Roman figure group (i.e. none, trypan blue, and cortisone, respectively), but none of the mice were injected with tumour ascites.

The survival time of each mouse that died in every group was recorded. When the mice in the Roman figure groups died the tumour ascites was removed and 1 ml. of this centrifuged at 2,400 r.p.m. for 45 minutes. The volume of the red blood cells at the bottom of the Wintrobe tube was taken as an estimate of the amount of blood present/ml. and recorded as a percentage of the total volume in the tube.*

### RESULTS

Table I shows the sex difference in the survival time and blood content of the tumour ascites in the mice in groups I, II and III, all of which died. The table gives the values for the total, male and female series, with the number of mice, the mean value with S.D., the S.E. of the actual difference between the male and female means, and the *t* and *P* values for this difference.

* One mouse in group II died of trauma the day after the injection of the tumour and was excluded from subsequent calculations.

TABLE I.—*The Sex Differences in Survival Time and Blood Content of the Tumour Ascites in the Three Groups*

| $x$ | Group | Series | $n$ | $\bar{x}$ | S.D.$\bar{x}$ | S.E. difference $\bar{x}_♂$ & $\bar{x}_♀$ | $t$ | $P$ |
|---|---|---|---|---|---|---|---|---|
| Survival time (days) | I | ♂+♀ | 30 | 11·03 | 2·683 | | | |
| | | ♂ | 15 | 10·8 | 2·186 | 1·013 | 0·4862 | 0·7>$P$>0·6 |
| | | ♀ | 15 | 11·3 | 3·253 | | | |
| | II | ♂+♀ | 29 | 12·24 | 3·332 | | | |
| | | ♂ | 14 | 12·9 | 2·901 | 1·33 | 0·9773 | 0·4>$P$>0·3 |
| | | ♀ | 15 | 11·6 | 4·188 | | | |
| | III | ♂+♀ | 30 | 14·93 | 4·528 | | | |
| | | ♂ | 15 | 11·8 | 3·571 | 1·233 | 5·077 | 0·001>$P$ |
| | | ♀ | 15 | 18·06 | 3·17 | | | |
| Blood content of ascites (vol. %) | I | ♂+♀ | 30 | 4·3 | 4·638 | | | |
| | | ♂ | 15 | 4·4 | 5·144 | 1·736 | 0·0806 | $P$>0·9 |
| | | ♀ | 15 | 4·26 | 4·331 | | | |
| | II | ♂+♀ | 29 | 1·717 | 4·286 | | | |
| | | ♂ | 14 | 2·036 | 3·782 | 1·702 | 0·2974 | 0·8>$P$>0·7 |
| | | ♀ | 15 | 1·53 | 5·304 | | | |
| | III | ♂+♀ | 30 | 1·216 | 2·616 | | | |
| | | ♂ | 15 | 0·9 | 2·569 | 0·9806 | 0·6424 | 0·6>$P$>0·5 |
| | | ♀ | 15 | 1·53 | 2·798 | | | |

The number of mice in each group and series is shown, the mean value for the factor under consideration ($x$) with its S.D., the S.E. of the actual difference between male and female means, and the $t$ and $P$ values for this difference.

The mean survival time for the total series was shortest in the control group I (11·03 days, S.D. 2·683), and longest in the cortisone treated group III (14·93 days, S.D. 4·528). The vitally stained group II fell between these two extremes (12·24 days, S.D. 3·332). The sex difference within the groups was not statistically significant in groups I and II (0·5 and 1·3 days respectively), while it was highly significant in group III (6·26 days, 0·001 > $P$). The mean blood content of the tumour ascites was highest in the control group I (4·3 per cent, S.D. 4·638), and lowest in the cortisone treated group III (1·216 per cent, S.D. 2·616). Once again the vitally stained group II showed an intermediate value (1·717 per cent, S.D. 4·286). The sex differences were not significant.

TABLE II*a*.—*Comparison of the Differences in Survival Time Between the Three Groups*

| | Comparison between groups $x$ | and $y$ | Series | $n_x$ | $n_y$ | Difference between $\bar{x}$ and $\bar{y}$ | S.E. difference between $\bar{x}$ and $\bar{y}$ | $t$ | $P$ |
|---|---|---|---|---|---|---|---|---|---|
| Survival time (days) | I | II | ♂+♀ | 30 | 29 | 1·21 | 0·7891 | 0·1533 | 0·9>$P$>0·8 |
| | I | III | ♂ | 15 | 15 | 1·0 | 4·187 | 0·2388 | 0·9>$P$>0·8 |
| | I | III | ♀ | 15 | 15 | 6·76 | 1·172 | 5·768 | 0·001>$P$ |
| | II | III | ♂ | 14 | 15 | 1·1 | 1·204 | 0·9137 | 0·4>$P$>0·3 |
| | II | III | ♀ | 15 | 15 | 6·46 | 1·356 | 4·764 | 0·001>$P$ |

The difference in survival time between the groups for the total series, or for the male and female series separately when their difference is significant, is shown, with the number of mice used and the S.E., $t$ and $P$ values for the difference.

Table II*a* compares the differences in survival time between the three main groups, giving the difference between the groups for the total series, or for the male and female series separately when their difference is significant, with the number of mice used, the S.E., $t$ and $P$ values for the difference.

The difference in survival time of the mice in groups I and II, total series (1·21 days) is not statistically significant. In groups I and III this difference (1 day) is not significant for the male mice, but the difference for the female mice in these groups (6·67 days) is highly significant (0·001 > $P$). The difference between groups II and III for the male mice (1·1 days) is not significant, but that for the females (6·46 days) is highly so (0·001 > $P$).

TABLE II*b*.—*Comparison of the Differences in Blood Content of the Tumour Ascites Between the Three Groups*

| | Comparison between groups $x$ and $y$ | | Series | $n_x$ | $n_y$ | Difference between $\bar{x}$ and $\bar{y}$ | S.E. difference between $\bar{x}$ and $\bar{y}$ | $t$ | $P$ |
|---|---|---|---|---|---|---|---|---|---|
| Blood content of ascites (vol. %) | I | II | ♂+♀ | 30 | 29 | 2·583 | 1·161 | 2·224 | 0·05>$P$>0·02 |
| | I | III | ♂+♀ | 30 | 30 | 3·184 | 0·9705 | 3·281 | 0·01>$P$>0·001 |
| | II | III | ♂+♀ | 29 | 30 | 0·501 | 0·7416 | 0·675 | 0·5>$P$>0·4 |

The difference in blood content between the groups is shown for the total series, with the number of mice used, the S.E., $t$ and $P$ values for the difference.

Table II*b* compares the differences in blood content of the tumour ascites between the three main groups for the total series, giving the number of mice used, the S.E., $t$ and $P$ values for the difference. The difference between groups I and II (2·583 per cent) is significant (0·05 > $P$ > 0·02), while that between groups I and III (3·184 per cent) is highly so (0·01 > $P$ > 0·001). The difference between groups II and III (0·7416 per cent) is not significant.

The relationship between the survival time and the amount of blood in the tumour ascites in the three main groups is shown in Table III, which gives the

TABLE III.—*The Relationship Between the Survival Time and the Blood Content of the Tumour Ascites in the Three Groups*

| Group | Series | $n$ | $r$ | S.E.$r$ | $t$ | $P$ |
|---|---|---|---|---|---|---|
| I | ♂+♀ | 30 | −0·8381 | 0·2397 | 3·496 | 0·01>$P$>0·001 |
| | ♂ | 15 | −0·9831 | 0·2673 | 3·678 | |
| | ♀ | 15 | −0·8708 | 0·2673 | 3·258 | |
| II | ♂+♀ | 29 | −0·5125 | 0·1889 | 2·713 | 0·02>$P$>0·01 |
| | ♂ | 14 | −0·4658 | 0·2773 | 1·680 | 0·2>$P$>0·1 |
| | ♀ | 15 | −0·7591 | 0·2673 | 2·840 | 0·02>$P$>0·01 |
| III | ♂+♀ | 30 | −0·01574 | 0·2397 | 0·06567 | $P$>0·9 |
| | ♂ | 15 | −0·02207 | 0·2673 | 0·08258 | |
| | ♀ | 15 | −0·2633 | 0·2673 | 0·9851 | 0·4>$P$>0·3 |

The number of mice in each group and series is shown, and the correlation coefficient ($r$) with its S.E., $t$ and $P$ values.

number of mice in each group and series, the correlation coefficient ($r$), with its S.E., $t$ and $P$ values. In the control group I the amount of blood in the tumour ascites decreased with survival time. This negative correlation (—0·8381, —0·9831, —0·8708 for the total, male and female series, respectively) was highly significant in all cases (0·01 > $P$ > 0·001). These findings are in keeping with those in the author's previous experiment (Hartveit, 1961). In the vitally stained group II the negative correlation was greatly reduced (−0·5125, −0·4658, −0·7591, respectively), being significant for the total and female series ($0{\cdot}02 > P > 0{\cdot}01$). It was even further reduced in the cortisone treated group III (−0 ·01574, −0·02207, −0·2633) and as such was not significant.

With regard to the control groups 1, 2 and 3 ; none of the mice in group 1 died. One of the female mice in group 2 died 12 days after its first injection of trypan blue. One male and one female mouse in group 3 died after 21 injections of cortisone.

### DISCUSSION

In this experiment there are three factors to be considered : the tumour, the relationship of the mice to the tumour and the interrelationship of the mice themselves.

As regards the tumour, Klein and Révész (1953) have shown that provided a sufficient tumour cell dose is used Ehrlich's ascites carcinoma can be expected to grow progressively, and the mouse to die within a time limit dependent on that tumour cell dose. The tumour cell dose used in the present experiment is comparable to that used in the author's previous experiment (Hartveit, 1961). The adult mice were taken from the same closed colony. On comparing the mice in group I of the present experiment to those in the previous experiment it is found that the actual difference in their mean survival time (0·53 days) is not significant ($0{\cdot}5 > P > 0{\cdot}4$). The difference in the mean blood content (1·7 per cent) is not significant either ($0{\cdot}2 > P > 0{\cdot}1$). In both cases the negative correlation between the survival time and the blood content of the tumour ascites is highly significant. Thus, although the tumour came from different transplant generations, there was no significant difference in the survival time or blood content, and the relationship between these two factors was unchanged. These facts support the view that the tumour can, in this case, be regarded as a relatively constant factor. In the present experiment all the tumour used came from one mouse, and this is an additional safeguard to the assumption that the tumour is a constant.

The mice were, of necessity, of different genetic make-up to the tumour, as Ehrlich's ascites carcinoma came originally from a heterozygous mouse (Snell, 1953). Therefore, one would expect that the mice would not accept transplants. But Ehrlich's ascites carcinoma is one of the so-called " non-specific " tumours that is said to contain fewer antigens than usual and so is less demanding than most in its choice of host (Barrett, 1958). However, a perfect fit between host and tumour cannot be expected. While the fit may be close enough to allow the tumour to grow the difference may show up in other ways—for example in the stromal reaction.

Thus, although the mice were not, as in Barrett's experiment referred to earlier (Barrett, 1942), sensitized beforehand with a foreign antigen, the tumour may possess tissue antigens that the mice lack. If they then react against this foreign protein their immunological response might be expected, on the basis of Barrett's findings, to be accompanied by haemorrhage into the tumour, i.e. by a change in the stromal reaction.

This hypothesis is based on the assumption that resistance to homotransplants is genetically determined. This has been shown to be true in the case of red blood cells (Cushing and Campbell, 1957) and normal tissues (Loeb and Wright, 1927 ;

Billingham, Brent, Medawar and Sparrow, 1954). The Mendelian nature of the genetic influences determining susceptibility to transplanted tumours has also been established (Little, 1956).

When a tumour is transplanted the tumour cells reproduce, the stroma is supplied by the host (Muir, 1951). In the case of Ehrlich's ascites carcinoma the stroma is represented by the ascitic fluid, which contains a variable number of white blood cells. In addition, in some cases, this stroma contains large numbers of red blood cells (Loewenthal and Jahn, 1932 ; Kun, Talalay and Williams-Ashman, 1951).

These red blood cells have appeared in the stroma either in direct response to the tumour cells, or in response to the mouse's reaction to these cells. If the first is the case we would expect that the amount of blood in the stroma would be constant for a given tumour cell dose. But the author has previously shown (Hartveit, 1961) that the amount of blood varies inversely with the survival time in mice receiving the same tumour cell dose. If the second is then true, the amount of blood should vary in accordance with the genetic dissimilarity between the mouse and the tumour.

Genetic dissimilarity is evidenced as resistance to transplantation (Snell, 1957). It has been shown that this resistance can be abrogated (Ludford, 1931 ; Andervont, 1936). The mice in the present experiment were treated in such a way as to abrogate their resistance to the transplantation of foreign tissue.

On the basis of the above reasoning one would expect that by supressing host resistance one would change the amount of blood present in the stroma : an increase indicating that the blood was an expression of the mouse's lack of resistance to the tumour, a decrease that it was an expression of its resistance. On the basis of Barrett's experiment (Barrett, 1942), one would expect a decrease.

The mice used were heterozygous. This does not affect their individual relationship to the tumour as discussed above, but different degrees of reaction are to be expected—those nearer to the tumour gentically reacting less against it. As the mice were from the same closed colony it is reasonable to assume that the genetic range in the three samples should be similar, and the results following abrogation of their natural resistance comparable.

All the mice receiving Ehrlich's ascites carcinoma in the present experiment were given the same dose of viable tumour cells. The dose used was chosen as it was known to give an ascitic tumour and a relatively short survival time. The dosage of trypan blue was based on that used by Andervont (1936), and the cortisone dosage on that used by Hobson (1960).

The results in the control mice show that the subcutaneous treatment alone will not influence the survival times of the experimental groups to any appreciable extent. Only one of the mice receiving trypan blue died during the course of the experiment and two of those given cortisone died towards its end.

The results in groups I, II and III show that, although all the mice died, their survival times and the blood content of their tumours differed considerably. Trypan blue did not alter the survival time of the mice but it did reduce the amount of blood in the tumour ascites. Cortisone increased the survival time of the female mice, and greatly reduced the amount of blood in the ascites in both sexes. In addition trypan blue, in part, and cortisone completely, abolished the negative correlation between the survival time and the amount of blood in the tumour ascites that was so strongly apparent in the control group.

Thus, by using methods claimed to reduce the natural resistance of the mice to tumour transplantation we have succeeded in increasing their survival time and decreasing their stromal reaction to the tumour. The blood content of the tumour must, therefore, be considered as an expression of resistance to Ehrlich's ascites carcinoma. We are then left with the paradoxical situation in which the mice with the greatest and most rapid local reaction to the tumour—indicating a type of resistance—die before those without such resistance. Those dying early died as a result of their immunological reaction to the homotransplant, while those surviving longer died as a result of their acceptance of the tumour that was able to grow progressively until it killed the host mechanically.

This finding that the blood content of the tumour is an expression of the resistance of the mouse to the tumour may be of practical use in the field of tumour immunity. In 1958 Barrett stated that antibodies " have eluded detection " in this field. Since then *in vitro* tests for haemagglutinins (Feldman and Sachs, 1957), the tanned erythrocyte technique (Finney, Byers and Wilson, 1960), complement fixation (Lund, 1958) and skin tests (Grace and Dao, 1958) have been reported to give positive results. However, most evidence of such immunity is circumstantial. Thus when an animal is immunized against a tumour the test of whether immunity has been produced is whether or not a transplant of that tumour will be able to grow in the host. Other methods involve the use of an immune serum from a foreign host (Flax, 1956). In this case the antibody is an antibody to a foreign protein, and is not dependent on the fact that the cells were tumour cells. With Ehrlich's ascites carcinoma the amount of blood in the ascites may prove to be a yard-stick by which it will be possible to measure induced as well as natural immunity to the tumour. Experiments in this direction are in progress.

It may be that this haemorrhagic reaction is a Shwartzman-like phenomenon. It has been suggested that the Shwartzman phenomenon may be a manifestation of an immune response (Thomas, 1954). Stetson (1955) has shown that injection of bacterial endotoxin can elicit haemorrhage in skin areas previously prepared by the intradermal injection of homologous or heterologous bacterial products, and compares the reaction to that following the injection of tuberculin in specifically sensitized rabbits. He postulates that both reactions involve a delayed type of allergy, that is accompanied by focal (haemorrhagic) reactions, and systemic reactions—following intraperitoneal injection of the endotoxin—that may be fatal. Lawrence (1956) has drawn analogies between this type of delayed sensitivity and homograft rejection. In the present experiment it has been shown that the haemorrhagic response occurring in some of the mice following transplantation of the tumour is dependent on their natural resistance to the transplant. This response can be likened to the Shwartzman phenomenon in that the mice were previously sensitive to the transplant and showed focal haemorrhage, and a severe systemic reaction leading to death, following a massive intraperitoneal dose of the incompatible material.

### SUMMARY

Three groups of 30 heterozygous mice were injected with Ehrlich's ascites carcinoma. Two of the groups of animals were treated with trypan blue and cortisone, respectively, in an attempt to abrogate their natural resistance to the tumour. The amount of blood in the tumour ascites was found to be less following these treatments. (Average per cent : Control—4·3, trypan blue—1·7, cortisone—1·2).

Thus the blood content of the tumour ascites can be regarded as an expression of the animal's reaction against, i.e. natural resistance to, the tumour. It was also shown that the survival time of the mice that did not react was greater than that of those which reacted against the tumour—suggesting that the latter died as a result of their immunological reaction to an overwhelming dose of foreign tissue. It is possible that this reaction is comparable to a Shwartzman-like phenomenon.

### REFERENCES

ANDERVONT, H. B.—(1936) *Publ. Hlth Rep., Wash.*, **51**, 591.
APITZ, K.—(1934) *Z. Krebsforsch.*, **40**, 50.
BARRETT, M. K.—(1942) *J. nat. Cancer Inst.*, **2**, 625.—(1958) *J. chron. Dis. (St. Louis)*, **8**, 136.
BILLINGHAM, R. E., BRENT, L., MEDAWAR, P. B. AND SPARROW, E. M.—(1954) *Proc. roy. Soc.* B, **143**, 43.
CUSHING, J. E. AND CAMPBELL, D. H.—(1957) 'Principles of Immunology'. London (McGraw Book Company, Inc.).
FELDMAN, M. AND SACHS, L.—(1957) *J. nat. Cancer Inst.*, **18**, 529.
FINNEY, J. W., BYERS, E. G. AND WILSON, R. H.—(1960) *Cancer Res.*, **20**, 351.
FLAX, M. H.—(1956) *Ibid.*, **16**, 774.
GRACE, J. T. AND DAO, T. L.—(1958) *Surg. Forum*, **9**, 611.
HARTVEIT, F.—(1961) *Brit. J. Cancer*, **15**, 336.
HOBSON, D.—(1960) *Brit. J. exp. Path.*, **41**, 251.
KARNOFSKY, D. A.—(1953) 'Experimental Cancer Chemotherapy'. In 'The Physiopathology of Cancer', ed. Homburger, F. and Fishman, W. H. New York (Paul B. Hoeber, Inc.), p. 635.
KLEIN, G. AND RÉVÉSZ, L.—(1953) *J. nat. Cancer Inst.*, **14**, 229.
KUN, E., TALALAY, P. AND WILLIAMS-ASHMAN, H. G.—(1951) *Cancer Res.*, **11**, 855.
LAWRENCE, H. S.—(1956) *Amer. J. Med.*, **20**, 428.
LITTLE, C. C.—(1956) 'The Genetics of Tumor Transplantation'. In 'Biology of the Laboratory Mouse', ed. Snell, G. D. London (Constable and Co. Ltd.), p. 279.
LOEB, L. AND WRIGHT, S.—(1927) *Amer. J. Path.*, **3**, 251.
LOEWENTHAL, H. AND JAHN, G.—(1932) *Z. Krebsforsch.*, **37**, 437.
LUDFORD, R. J.—(1931) *Brit. J. exp. Path.*, **12**, 108.
LUND, H. J. C.—(1958) *Acta path, microbiol. scand.*, **43**, 391.
MUIR, R.—(1951) 'Textbook of Pathology'. 6th ed., revised Cappel, D. F. London (Arnold and Co.), p. 280.
SCHREK, R.—(1936) *Amer. J. Cancer*, **28**, 389.
SNELL, G. D.—(1953) 'Transplantable Tumors'. In 'The Physiopathology of Cancer', ed. Homburger, F. and Fishman, W. H. New York (Paul B. Hoeber, Inc.), p. 343.
SNELL, G. D.—(1957) *Ann. N.Y. Acad. Sci.*, **69**, 555.
STETSON, C. A., Jr.—(1955) *J. exp. Med.*, **101**, 421.
THOMAS, L.—(1954) *Ann. Rev. Physiol.*, **16**, 467.
TOOLAN, H. W.—(1953) *J. nat. Cancer Inst.*, **14**, 745.